# Cultural Determinants of Body Image: What About the Menopausal Transition?

**DOI:** 10.3390/healthcare13010076

**Published:** 2025-01-03

**Authors:** Coralie Vincent, Alixe Ménard, Isabelle Giroux

**Affiliations:** 1 Interdisciplinary School of Health Sciences, Faculty of Health Sciences, University of Ottawa, Ottawa, ON K1N 6N5, Canada; amena069@uottawa.ca; 2 School of Nutrition Sciences, Faculty of Health Sciences, University of Ottawa, Ottawa, ON K1N 6N5, Canada; igiroux@uottawa.ca; 3 Institut du Savoir Montfort, Ottawa, ON K1K 0M9, Canada

**Keywords:** body dissatisfaction, culture, risk factors, women’s health, menopause, climacteric, aging, perceptions

## Abstract

Body image is an important aspect of psychological well-being that is influenced by several biological and psychosocial risk factors. Cultural determinants of body image include the patterns of shared beliefs, values, practices, and social norms within a group that can act as a lens through which a person perceives, compares, and evaluates their body. Women tend to experience higher rates of body dissatisfaction than men, with reproductive milestones such as puberty, pregnancy, and postpartum being windows of vulnerability for body image concerns. The menopausal transition is another reproductive stage of women’s lives that involves major physical changes, psychological challenges, and social pressures that can impact body image negatively. However, the literature on the influence of cultural determinants on the body image of menopausal women is limited. Therefore, this perspective review paper aims to discuss the potential role of cultural determinants in influencing body image satisfaction in women undergoing the menopausal transition. To this end, the relationships between different cultural perspectives and body image around the world are first discussed. Sociocultural influences on body image throughout women’s lifespan and reproductive stages are then presented. Finally, cultural perspectives on menopause and aging and their potential influence on the body image of menopausal women are explored. This paper underscores the importance of considering culture and sociocultural factors when studying body image and highlights the need for further research on the cultural determinants of body image during the menopausal transition.

## 1. Introduction

Body image is commonly defined as a person’s perception, thoughts, feelings, and attitudes towards their own body [1]. It is a multidimensional construct that comprises the emotional importance attributed to one’s own appearance as well as self-perceived attractiveness, physical appearance, and body functioning (i.e., how well one’s body meets functional expectations) [2]. It is often assessed through the level of concern, satisfaction, confidence, or acceptance towards the body or through behavioural tendencies such as altering, concealing, or tending to one’s own body [3,4]. Body image satisfaction is a major contributor to global psychological health [5,6,7]. Having a positive body image is associated with better overall quality of life [6], greater sexual well-being [8], and higher adherence to a variety of health-promoting behaviours [9,10]. Contrastingly, body image concerns are associated with a spectrum of mental health disorders including depressive symptoms [7,11,12], anxiety [5,13], and low self-esteem [14]. Body dissatisfaction is also widely recognized as the most robust predictor and contributor to disordered eating behaviours [15,16,17].

Women tend to be disproportionately affected by body dissatisfaction compared to men [18], which is in part due to the overwhelming social pressures and unrealistic societal norms of beauty that are imposed onto this gender. Disturbingly, these gender differences have been reported in children as young as eight years old [19], with women showing consistently higher vulnerability to body dissatisfaction across their lifespan [20]. This is in part due to the sociocultural expectation for women to conform to the “thin ideal”, a socially constructed and culturally reinforced standard of beauty that is heavily promoted across media and social discourse in Western cultures [21,22].

The vast majority of research on body image disturbances revolves around teens (10–18 years old) and young adults (19–25 years old), commonly showing rates of body dissatisfaction exceeding 50% of participants [2,4]. In recent decades, growing evidence has emerged demonstrating similarly alarming rates of body dissatisfaction in middle-aged or midlife (40–60 years old) and older (over 60 years old) women, with several studies reporting that 40 to 80% of midlife women struggle with body image concerns [23,24,25,26,27]. In fact, body dissatisfaction has become so ubiquitous among women and girls, particularly in Western societies, that it has been described as “normative discontent” [28,29].

The study of body image cannot be separated from its cultural context due to the paramount influence of sociocultural factors on the development of body satisfaction and dissatisfaction [21]. Cultural determinants of health refer to the social, cultural, and environmental factors that influence individuals’ health behaviours, perceptions, and outcomes [30]. While the study of racial and ethnic differences is often used as a proxy to investigate cultural differences, culture is a much more complex concept, encompassing the patterns of shared beliefs, practices, values, traditions, and social norms within a group and acting as a lens through which both the individual and the collective perceive and experience the world [31]. Culture has also been described as an internalized and shared schema that sets out the unwritten expectations within a cultural group [32]. In the context of body image, some impactful components of culture include the societal norms of beauty, media influences, and social pressures from peers, family, and romantic partners [16,33]. As the context within which body image develops, cultural environment plays a key role in moulding our perception of and feelings about our bodies. It is not only the environment through which beauty ideals are conveyed but also the social setting in which we engage in appearance-related comparisons [22].

Over women’s lifespan, puberty, pregnancy, and postpartum have been identified as windows of vulnerability for the development or exacerbation of body image disturbances [34,35,36,37,38,39]. Indeed, these reproductive milestones involve a complex storm of physical, psychological, and social changes that can deeply affect women’s sense of identity and satisfaction with their body, as they try to navigate the often narrow and unrealistic expectations of beauty imposed during these critical life stages [2]. While there is growing evidence of the concerning levels of body dissatisfaction among midlife women [40,41,42], literature pertaining to the menopausal transition as a particular period of interest remains limited [43]. Yet, this transition bears many similarities to other reproductive milestones of women’s lives. Menopause is a biological phenomenon related to the female sex that commonly takes place around the age of 50 [44], although the median duration of symptoms spans over 7.4 years [45], and refers to the cessation of menses and reproductive potential caused by a decline in estrogen levels and follicular activity [46]. The menopausal transition commonly spans several years prior to and after the last menstrual period and includes the late premenopausal, perimenopausal, and early postmenopausal stages during which women can experience a myriad of physiological and physical changes, an increased risk of mental health challenges, and social and societal pressures related to menopause and aging (premenopause: less than 3 months of change in the frequency of menses; perimenopause: at least 3 months of change in the frequency of menses and less than 12 months of amenorrhea; postmenopause: begins after 12 months of amenorrhea) [43,47]. 

Previous literature has highlighted important cultural differences in body image satisfaction and the perceptions of menopause and aging around the world [32,48]. However, studies exploring how cultural determinants specifically shape the body image of women during the menopausal transition are scarce. With approximately 47 million women reaching menopause each year [49], the study of women’s psychological and physical well-being throughout this complex transition is of great importance. Therefore, this perspective review paper aims to discuss the potential role of cultural determinants in influencing body image satisfaction during the menopausal transition. To this end, the influence of different cultural perspectives on body image globally will first be discussed. Sociocultural influences on body image throughout women’s reproductive stages will then be presented, before discussing different cultural perspectives on aging and menopause and their potential influence on the body image of menopausal women. This paper uses a lifespan approach to highlight the complex intersection of biological, psychological, social, and cultural factors related to women’s body image satisfaction. It also emphasizes the need for further research on body image during the menopausal transition and the importance of cultural determinants in shaping body satisfaction during this significant stage of life.

### Sex and Gender Considerations

The study of body image involves many important psychosocial implications that are closely tied to gender as a social construct. Meanwhile, biological phenomena like pregnancy and menopause are typically exclusive to female sex biology. Nonetheless, some female sex individuals who may experience pregnancy and menopause identify as other genders (e.g., non-binary, trans man). Unfortunately, literature on the experience of pregnancy and menopause in these individuals is scant [50,51], particularly when it comes to associations between cultural determinants and body image. While this paper uses the term “women” throughout most of the text, it should be noted that the sections focused on the experience of pregnancy, postpartum, and menopause in relation to culture and body image typically refer to cis-gendered women, as this is almost exclusively the population included in the cited literature. While many of the presented social and cultural determinants of body image can be relevant to an array of gender-diverse individuals, the determinants presented in relation to pregnancy and menopause in this paper are only representative of the experience of cis-gendered women.

## 2. Cultural Determinants of Body Image

There are many ways in which cultural determinants can affect body image satisfaction. At the societal level, culture can mould the standards of beauty to which people compare themselves and can shape the shared values and belief systems that influence people’s propensity to care about and strive towards those standards [52]. Societal pressures regarding beauty ideals are largely promulgated through the media (e.g., movies, television (TV), advertisement, magazines, social media) and have been repeatedly demonstrated to be an important risk factor for body dissatisfaction [16,52,53,54,55]. The rise of social media platforms has recently intensified the regular exposure to idealized images, making these standards appear both ubiquitous and mandatory [52]. On the social and community levels, shared beliefs and social norms relating to beauty and body image, including those presented through the media, may be passed along through conversations with, or comments from, peers, colleagues, family members, and romantic partners [33]. Importantly, the concept of social pressure does not simply relate to direct comments or teasing about appearance from one individual to another, but it rather encompasses all discourse (self-directed or aimed at others) about appearance, body weight, and body shape (e.g., a parent expressing discontent about their own weight in front of their child) [56,57,58,59,60]. Notably, a key factor in the development of body dissatisfaction is not only being exposed to the thin ideal but also personally internalizing this standard of beauty [16]. Thin-ideal internalization refers to the level to which an individual “buys into” the societal ideal of thinness, such as by defining their own worth, value, and attractiveness in relation to this ideal and making efforts to attain it [20,61]. Indeed, in the presence of the same media and social pressures, women with higher levels of thin-ideal internalization as well as higher levels of appearance comparison (i.e., tendency to compare their appearance to that of others) are at higher risk of developing body dissatisfaction [16,53].

The social and societal aspects of culture, in combination with other personal factors such as body mass index (BMI), can interact to influence people’s perceptions of their bodies in different ways across the world. Most research on cross-cultural differences in body satisfaction has been conducted on adolescents and young adults [22,32,62]. Indeed, a 2024 systematic review on cultural differences in body image found that only 9 out of 54 total studies included participants aged above 35 years old, with no study solely focusing on any population beyond that age [32]. Still, the observed trends in such reviews can help highlight the importance of cultural determinants for body image satisfaction and point to cultural differences that potentially extend to older populations such as menopausal women.

## 3. Body Image Across the World

### 3.1. Western Cultural Perspective

The “Western World” typically refers to North American countries (i.e., United States [US] and Canada), Western Europe (e.g., the United Kingdom [UK], France, Germany, Italy, Spain, Scandinavia, etc.), Australia and New Zealand. For women and girls, the beauty standards propagated among Western culture are highly characterized by the thin ideal and are often unattainable for the average individual, leading to widespread feelings of inadequacy and dissatisfaction [32,52]. In general, Western culture positions thinness as the most important marker of beauty, with media portrayals of female bodies sending constant pressures through direct (e.g., extreme thinness of fashion models; “fat jokes” in TV shows; magazines glamourizing extreme weight loss) and indirect (e.g., near totality of lead female romantic roles being played by thin women; overweight female movie characters being displayed as “mean” or “ugly”) reminders [56,63,64,65,66]. Along with thinness, Western beauty ideals focus on a youthful appearance as a requirement for beauty, leaving women on an endless chase to fight against their biological clock and appear younger than their natural age [67].

Nonetheless, beauty ideals are not a stagnant concept, which has recently been demonstrated by a shift in Western culture from the original “skinny” ideal to a new “fit” ideal, especially in younger generations of women [68]. While the “fit” beauty ideal still encourages thinness and low body fat, it puts more emphasis on being “strong” and “healthy”, which some have proposed is a way for diet culture (i.e., set of beliefs and practices that promote weight loss and thinness) to reframe itself through a “health-focused” lens, although its negative influence on body image likely persists through the perpetuation of weight stigma [68,69].

The incessant social and societal pressures present in Western cultures can lead women and girls to be dissatisfied with their appearance and weight, even when they have a “healthy” BMI [2,22,70]. Indeed, rates of body dissatisfaction across Western countries are mostly accounted for by a desire to be thinner, which is internalized at an alarmingly young age [2,7]. A longitudinal study of over 7000 children in the US revealed that 63.2% of girls expressed concern about gaining weight by age 13 [71], while a cross-sectional school-based Canadian study of 1515 preadolescent students found that 50.5% of girls (compared to 35.9% of boys) reported wanting a thinner body shape [72]. Meanwhile, studies on middle-aged and older women performed in North America show that this concern with body weight persists through the years, with one study of 405 participants showing that over 70% of US women (42–52 years old) reported being “somewhat unsatisfied” or “unsatisfied” with their weight [7], whereas a recent Canadian study (*n* = 1083) found that 90% of women aged 50–69 years old desired to lose weight [26].

With a few exceptions that will be discussed in the next section, most cross-cultural studies find that women’s levels of body dissatisfaction are higher in Western compared to non-Western cultures [32,73], with the US showing the highest levels of body dissatisfaction when compared to other Western countries [22]. For example, one cross-cultural study of young-adult women living in Italy, Australia, England, and the US found that appearance-ideal internalization and appearance pressures were the highest in the US and the lowest in Italy [74]. Similarly, studies have shown that US college students tend to be more dissatisfied with their body compared to both their German and Spanish counterparts [75,76]. A multinational examination of fat-phobia (i.e., anti-fat attitudes and biases) in the US, Canada, Iceland, Australia, and Hawaii found consistently high self-reported levels of anti-fat attitudes in both the adult and student samples of all regions, yet again highlighting the pervasiveness of the thin ideal across Western countries [77].

Western culture rewards conformity to the thin–young ideal via increased social status, value, and power [20]. Conversely, individuals that fail to meet beauty standards may be faced with bias, stigma, and discrimination in social contexts, for work opportunities, and in the healthcare system [78,79,80,81,82], which helps explain the high levels of distress felt around body image and weight in Western societies. It is also believed that certain shared values of Western culture such as individualism, consumerism, autonomy, external validation, the importance of appearance, and the pursuit of personal success, which are particularly strong in the US, could exacerbate people’s feelings of inadequacy and need to strive towards beauty ideals and could be partly responsible for the higher body dissatisfaction in the US compared to other Western countries [22,52].

Importantly, cultural differences are also found within regions and subcultures of Western countries and can be investigated through racial/ethnic differences within one country or through other cultural differentiators such as religious affiliation [22]. For instance, differences have been reported in the body satisfaction and body size perception of Black American and Caucasian women living in the US [22]. Less body dissatisfaction and a preference for a slightly larger or curvier body shape are generally found in Black Americans compared to their Caucasian counterparts, which could in part be explained by a cultural preference for curvier female figures in Black American culture [83,84,85]. However, these results were not consistently observed among all studies [7].

Another example of within-country cultural differences in body image is a cross-sectional study of Muslim and non-Muslim women (*n* = 201) performed in the US which found that Muslim women wearing non-Western clothing and a head veil (also known as hijab or head scarf) were significantly less likely to express a drive for thinness or pressure to attain the thin ideal compared to women wearing Western dressing (i.e., standard American clothes), which the authors proposed could indicate a potential protective role of religion for body image satisfaction within the context of Western culture [86]. In fact, a 2019 study of 345 adult Australian women found that participants who engaged with a formal religion or with general spirituality held a more positive body image, which was mediated by more gratitude, a de-emphasis on external appearance (higher intrinsic value), and less self-objectification [87]. Still, the relationship between religion and body image is complex, with different aspects of religiosity being associated with both positive and negative body image across the literature [88,89].

### 3.2. Beyond Western Culture

Beauty standards and cultural norms related to the pursuit of beauty in non-Western cultures vary considerably from those of the West. For example, one study conducted across six countries recruited Black Nigerian women, Chinese women, and Western women (from the UK, USA, Canada, and Australia) and found that Nigerian women reported the highest body appreciation, while Western women reported the lowest [73]. This study also highlighted the role of lower thin-ideal internalization and lower perceived sociocultural pressure in explaining the higher body appreciation levels found in non-Western women [73]. Another large-scale study investigated female body dissatisfaction across 26 different countries and found that South and North American women displayed significantly more body dissatisfaction than women in Western Europe, Eastern Europe, Southeast Asia, South and West Asia, and Oceania [90], hinting towards the potential of non-American cultures to foster a more positive body image.

African, Middle Eastern, and Oceanic cultures are often found to hold different beauty ideals than the West when it comes to body weight and shape [32]. Indeed, residents of Malaysia and Pacific Islander countries such as Fiji, Tonga, and Samoa often prefer larger bodies and report less body dissatisfaction when compared to Australians, as their traditional cultures and religions typically value large and muscular female bodies [32,91,92,93]. African women, including in some African Middle Eastern countries, also tend to be more accepting of higher body weights, with certain regions and subsequent cultures even preferring larger bodies [90,94,95]. Moreover, one study comparing Arab immigrants to Germans living in Germany found that Arab immigrants held larger body image ideals compared to German participants [94]. However, globalization has largely expanded the reach and influence of the Western thin ideal around the world, infiltrating many countries traditionally shown to prefer larger bodies [32,95]. For instance, Swami et al., 2010 reported that Malaysian and South African participants from rural and low-socioeconomic regions (i.e., Sabah and KwaZulu-Natal) rated heavier female figures more positively, whereas participants living in urban, high-socioeconomic areas (Kuala-Lumpur and Cape Town) in the same countries rated slender figures more positively [90]. Similarly, a systematic review of body image perception among African immigrants in Europe found that ethnic groups living in great isolation or with low income continue to prefer larger bodies, while ethnic groups living in urban areas are moving towards a Westernized beauty ideal and prefer underweight and normal body weights [95].

Certain Asian countries such as China and India also generally tend to have higher body satisfaction levels than Western cultures [32]. One study showed that Chinese adolescents showed less endorsement of the thin ideal compared to American students and that they were less likely than American students to hold negative stereotypes towards obesity [96]. Nonetheless, one of the most prominent trends identified across Asian cultures is that highly modernized countries such as Japan and South Korea have the highest levels of body dissatisfaction, typically being even higher than those in Western cultures, including the US [32,97,98,99]. In fact, one cross-cultural study of Chinese, Malaysian, Australian, Tongan, Indigenous Fijian, and Japanese adolescents found that despite possessing one of the lowest mean BMIs, Japanese adolescents reported the highest levels of body dissatisfaction and reported some of the highest levels of media influence on their body image [100].

It is possible that some shared values and beliefs of Asian cultures may have a role in explaining such differences in body satisfaction. For instance, a mixed-method study by Ghotbi et al., 2017 found that Japanese and Chinese students had overall negative attitudes towards cosmetic surgery, while Korean students had overall positive attitudes towards these same procedures [101]. Their qualitative investigation of this phenomenon revealed that the negative views of Japanese students towards cosmetic surgery came from the values of modesty, naturalness, concern for safety and health (rather than appearance), respect to their parents, and individuality, similar to the Chinese students who mentioned their value of respect to their parents and the importance of preserving their inherited characteristics [101]. It could be argued that such values of community, family, collectivism, and respect contrast with the more individualistic and success-driven values of the West [52]. Indeed, the Korean students mentioned the values of beauty, utility (e.g., for job opportunities and social relations), autonomy, and popularity as a justification for their positive attitude towards cosmetic surgery, which highlights how modernization and globalization may have increased the influence of Western values and beliefs in South Korea. These findings highlight how different beauty standards, as well as different cultural values and belief systems, can influence people’s beauty satisfaction and views of beauty around the world and underline differences between and within Western and non-Western cultures. Notably, there are many other aspects of cultural beauty standards that were not discussed here (e.g., skin tone, facial features) [102,103], as they are less likely to be relevant when considering body satisfaction during menopause and were thus considered beyond the scope of this paper.

## 4. Sociocultural Factors and Body Image Throughout Women’s Reproductive Stages

There is limited evidence on the sociocultural determinants of body image satisfaction during the menopausal transition. Nevertheless, a lifespan approach can be used to highlight important sociocultural influences on body image throughout women’s lifespan and other reproductive milestones such as puberty, pregnancy, and postpartum, in order to inform and compare how such social and cultural factors may influence women’s body image in various ways within the context of menopause.

### 4.1. Puberty

Puberty refers to the period during which adolescents gain reproductive capability and experience a myriad of physical, social, and psychological changes [56]. Entering puberty is associated with an increased risk of experiencing psychological issues such as depressive symptoms, anxiety, stress, and poor self-esteem [104,105]. Meanwhile, physical changes during puberty typically include an increase in body fat that is faster and more obvious in girls than in boys, leaving teen girls to experience greater concern over their weight and appearance [56,72]. In fact, several studies have shown that earlier pubertal onset is a predictor of more body dissatisfaction in girls and that body dissatisfaction increases with more advanced stages of pubertal development [36,106,107]. Still, the complex relationships between physical appearance and body dissatisfaction during puberty largely depend on other social and societal influences that establish the context within which girls conceptualize and compare their bodies [33].

Media pressure is one of the key factors influencing body satisfaction during puberty, as teenagers experience heightened appearance comparisons and high vulnerability to the internalization of beauty ideals [56]. A longer time spent being exposed to the media is associated with a higher risk of body image concerns in teens [108,109]. This is particularly true for appearance-oriented TV and social media platforms that generally showcase Western-influenced, highly curated, and idealized female bodies. Puberty also marks the transition when teenage girls may start to be and feel sexualized by both society and their social circles [56]. Exposure to sexualizing content and internalized sexualization have been associated with poorer body image and greater body shame in teenage girls [110,111].

Social influence from parents also has a major role in affecting the body satisfaction of teenagers, with positive parent–adolescent relationships even showing an ability to moderate the negative influence of the media on body image [112]. Conversely, negative parental comments or pressures about either their own or their child’s body and eating habits can lead to lower body satisfaction in teenagers [113,114,115]. In fact, there appears to be a generational component to body dissatisfaction in women, with research on 91 pairs of mothers and college-aged daughters showing that mothers with a greater internalization of media messages about thinness were most likely to have daughters with eating pathologies [116]. Furthermore, negative feedback from mothers, mothers’ disapproval of daughters’ figures, and mothers’ eating behaviours and attitudes as perceived by their daughters are all predictors of daughters’ body image [116]. Notably, ethnicity may play a role in the parent’s perception of their child’s weight. A 2019 systematic review found that while ten studies reported no association between ethnicity and parents’ misperception of children’s weight status, seven studies indicated that Caucasian parents were less likely to misperceive their children’s weight status compared to parents of other ethnicities (e.g., Black, Hispanic, South Asian), hinting that parents of other ethnicities could be less critical of their children’s overweight [117]. Still, one study of 186 Black immigrant mothers of Sub-Saharan African and Caribbean origin living in Canada found that mothers were more likely to perceive their overweight daughters as being overweight than their overweight sons [118].

As parental influence may vary across cultures, puberty and adolescence also mark a developmental stage where influence from peers typically begins to play a more prominent role, with reliance on friends for support and approval increasing drastically [56]. Concern about finding friends, attracting romantic partners, or participating in sports with peers may heighten teens’ orientation towards their body and appearance and lead to increased social comparison and desire to be accepted [56,119]. Indeed, lower acceptance by peers has been associated with poor body image among adolescent girls [119]. Additionally, appearance-related teasing from both peers and family members is a significant predictor of body dissatisfaction in teens, with 21–42% of adolescents reporting having experienced appearance-related teasing from at least one parent [56,120,121]. Overall, these findings highlight how beauty standards set at the societal level are passed along and shared through different environmental and social channels and can combine with the physical and psychological challenges of puberty to bring increased body dissatisfaction in adolescent girls.

### 4.2. Pregnancy and Postpartum

The perinatal period (i.e., pregnancy and postpartum) is another important reproductive stage of some women’s lives that involves large hormonal variations as well as a higher risk of psychological issues (e.g., depressive symptoms) and that bears many similarities to puberty in terms of the sociocultural factors influencing body dissatisfaction [122,123,124]. Many studies have reported that the physical changes developed as part of pregnancy and postpartum (e.g., weight gain, stretch marks, acne, “saggy” belly and breasts) are a source of increased body dissatisfaction, particularly in postpartum women, as they tend to diverge from the thin, fit, and youthful beauty ideal held by many cultures [125]. Still, pregnant women report conflicting feelings about their changing bodies, experiencing both pride about their body’s capabilities and shame about their postpartum appearance [37,126].

Media pressures related to women’s bodies and the thin ideal continue to hold a central role in affecting body satisfaction during the perinatal period [122], although there is now the addition of media pressures specific to the depiction of pregnant and postpartum women. Indeed, in qualitative studies, perinatal women report finding media depictions of pregnant and postpartum women to be highly idealized, unrealistic, and much different from their own complex experience during this challenging time, which can increase their feelings of inadequacy and body dissatisfaction [126,127,128]. There is also a large subset of the media dedicated to glamourized depictions of “bouncing back” after childbirth, which encourages postpartum women to strive towards returning to their pre-pregnancy weight as fast as possible [128,129]. Still, quantitative studies show that the relationship between media pressures and body dissatisfaction continues to be strongly affected by thin-ideal internalization and appearance comparison in perinatal women [125,130].

A new form of social pressure arising during pregnancy is that of health professionals, with qualitative studies indicating that some women report feeling pressure from their care provider to lose weight or to not gain too much weight during and after pregnancy [60,125]. While pressures from family and peers can still have a negative influence on body image in perinatal women [122], some qualitative reports have identified a potential protective role of peers and family when they display supportive and understanding attitudes towards pregnancy-related bodily changes [126].

Additionally, a recent systematic review of qualitative studies identified a recurrent theme of postpartum women having to negotiate their different roles and identities within society, with the transition into the mothering role as well as pregnancy-related changes being perceived as incompatible with women’s other roles as a wife, as a sexually attractive woman, as a working woman, and, ultimately, as their core self (i.e., their inner self or personal identity separate from other societal roles and expectations) [125]. Participants in one qualitative study also mentioned that postpartum appearance pressures felt gendered, with a high contrast between the unrealistic and idealized representations of new mothers compared to new fathers (even though mothers are the ones experiencing the physical impacts of pregnancy and childbirth) [126].

Cross-cultural studies comparing body satisfaction in perinatal women from different countries and cultures were hard to identify [32]. Still, the trends mentioned in Section 1 of this paper (coming mainly from the adolescent and young adult literature) may extend to the perinatal period. For instance, a few studies from the US echoed findings on racial and ethnic differences in young adults by showing that although African American women tend to gain more gestational weight and are more likely to retain weight gain postpartum [131], they generally express significantly lower levels of body dissatisfaction throughout and after pregnancy compared to Caucasian women [132,133].

### 4.3. Menopause and Midlife

The menopausal transition is a complex stage that marks the end of women’s reproductive life [46]. Again, many similarities can be found between the challenges of puberty and the perinatal period and those of the menopausal transition, including the increased risk for anxiety and depressive symptoms [134,135]. In fact, body dissatisfaction has been found to be a predictor of anxiety and depression in early postmenopausal women [5]. Menopause is also a period of intense physical changes, usually involving an accelerated increase in fat mass (particularly trunk and visceral fat mass); a decrease in muscle mass and bone mineral density; a loss in skin elasticity; and changes in hair texture, color, and distribution [136,137,138,139]. These changes are largely interpreted by menopausal women as straying away from the thin and youthful beauty ideal and often bring an increase in psychological distress and body dissatisfaction [5,7,67,140]. In qualitative studies, weight gain and shape changes are the most commonly reported causes of body dissatisfaction in menopausal women [42,140], echoing what is reported during puberty, pregnancy, and postpartum, although menopausal women are more likely to report that their concern over weight is for both appearance and health reasons [42,141]. Similarly to the perinatal period, menopausal women often find themselves fighting their own body, as they report experiencing social and societal pressures to resist and fight against the natural physical changes caused by menopause and aging [67,140,142]. In addition to appearance changes, most menopausal women will experience some form of menopausal symptoms, which include vasomotor (e.g., hot flashes, night sweats), somatic (e.g., fatigue, body aches, joint pain, sleep problems), urogenital (e.g., vaginal dryness, sexual dysfunction, frequent urination), and psychological (e.g., mood changes, irritability, brain fog) symptoms [46,143,144]. Interestingly, a 2023 systematic review of quantitative studies found that menopausal symptoms may be a key driver of body dissatisfaction in menopausal women [43].

Some authors have pointed to midlife as a period of increased social stressors for women, as they may face challenges such as a divorce, widowhood, a return to the dating game, empty-nest syndrome, high pressures at work (often being at the height of their professional careers), and a care-taking role for their children, older parents, and other family members [67]. Meanwhile, most sociocultural risk factors of body dissatisfaction during puberty and the perinatal period also seem to predict body image concerns in midlife women [145]. Indeed, one review identified several cross-sectional studies showing that thin-ideal internalization; self-objectification; perceived pressure from peers, family, and the media; and weight-related teasing were also significantly associated with body dissatisfaction in middle-aged women [145]. Interestingly, while pregnant women are often depicted positively in the media, in a glowing—albeit glamourized and unrealistic—way, menopausal women are usually portrayed in a negative light, presented as old, obsolete, irritable, “sexless”, and unattractive [146], highlighting an intersection of ageist and sexist stereotypes which could lead women and the general population to hold more negative views towards menopause [147,148]. This also contrasts with the sexualization undergone by teenage girls in the media, as menopausal women tend to be “de-sexualized” instead [146], although it could be argued that both experiences can negatively affect body image. These unfavorable media representations of menopause, which are more typical of Western culture, could exacerbate feelings of inadequacy, aging anxiety (i.e., negative feelings and fears about growing old), and body image dissatisfaction in menopausal women [149,150]. In fact, menopause is associated with an increase in aging anxiety [151], a phenomenon that is also reported across qualitative studies on women’s experience of menopause [140] and is a significant predictor of body dissatisfaction in midlife women [145].

During menopause, social pressure can also arise from relationships with romantic partners, with multiple studies finding a negative influence of menopause on the relationship and sexual satisfaction of menopausal women and their partners [152,153,154,155]. For example, one qualitative study found that men often had poor knowledge of menopause, misconceptions around the severity of symptoms, and had difficulty dealing with their wives’ menopause-related mood changes, which led to increased conflict in the relationship [152]. A qualitative study that conducted group discussions with a total of 81 Macedonian women living in Australia even identified a recurring theme of Macedonian men regarding their wives differently after menopause, sometimes treating them as “non-sexual” [156], which could in part be influenced by the abovementioned “de-sexualization” of menopausal women in the media.

Notably, similar to the identity challenges reported by postpartum women, many menopausal women report conflicting feelings about their aging appearance, as their reflection in the mirror often clashes with their perceived “real” or “core” self [42,140,157]. It could also be proposed that, similar to what was reported by postpartum women [127], the expectations of beauty through the aging process are gendered, with more pressure imposed on middle-aged women to retain a youthful appearance, whereas middle-aged men appear to retain most of their social and aesthetic value even when showing physical signs of aging. Nonetheless, qualitative investigations into women’s experience of the menopausal transition have exposed several perceived positive aspects of this stage of life, including some women reporting feeling a sense of increased wisdom and liberation from the burden and responsibility associated with reproductive life, which may help foster more positive attitudes towards the body during this transition [158].

Interestingly, there is evidence that some physical activity and psychosocial interventions, including therapeutically based group-format interventions, can lead to improvements in body image in midlife women [159]. For instance, one randomized controlled trial in middle-aged women reported significant improvements in body satisfaction after participants attended eight weekly educational sessions on topics including age-related appearance changes, self-worth, body acceptance, the importance of self-care, mindful eating, and behavioural strategies to reduce dieting and disordered eating [160].

## 5. Cultural Perspectives on Menopause: Potential Implications for Body Image

In the context of this paper, few studies could be identified on cross-cultural differences or cultural influences on the body image of midlife women in relation to the menopausal transition. Searches in the Medline, Embase, and PsycInfo databases using the MeSH terms “Menopause” and “Body image”, as well as a variety of MeSH terms and keywords surrounding cultural determinants (e.g., ethnic identity, racial groups, cultural/sociocultural factor, cross-cultural differences/comparison, culture, cultural norm, etc.), identified a total of only 37 non-duplicate records, with only 3 original studies found to be relevant to this review, all of which used a qualitative methodology [142,157,161]. In a recent systematic review of 18 quantitative studies on the associations between menopause and body image, 15 studies were performed in Western countries (US, UK, Australia, Austria, Switzerland, France, and Poland), while the other 3 came from the Middle East (Lebanon, Turkey, and Israel) [43]. Of these, only four studies reported the ethnicity of their participants, and none reported whether ethnicity moderated the reported relationship between menopause-related factors (i.e., menopausal stages, menopausal symptoms, hormonal factors) and body image [43]. Moreover, the high heterogeneity in the methods and tools used across studies and countries makes it difficult to perform cross-country comparisons of body image scores. One of the first and only multiracial quantitative studies of body image in middle-aged women is the Study of Women’s Health Across the Nation (SWAN) published in 2014, which reported no significant differences in body dissatisfaction between African American and Caucasian US women aged 42–52 years old, although they did not report whether there was an association between menopausal status and body image or whether race was a moderator of such an association [7]. More recently, a 2019 qualitative study that conducted interviews and focus groups with a multiracial group of 39 peri- and postmenopausal women (45–60 years old) found that Black women were more likely to discuss feeling confident than White women and highlighted that midlife women’s body image concerns had an important influence on their sexual satisfaction [161].

Meanwhile, cross-cultural studies on the general physical and psychological experiences of menopause could inform potential cultural and ethnic differences in body image during this stage. For example, there are large differences in the prevalence, severity, and types of menopausal symptoms experienced across different regions of the world [162]. Indeed, women in African and Asian countries tend to report a lower incidence of vasomotor symptoms compared to North American and European populations, whereas Asian women tend to report the highest rates of menopause-related joint and muscle pains [162,163,164]. In a study of ethnic differences in the US, Japanese and Chinese women reported the least number of menopausal symptoms, while African American women reported more vaginal dryness and vasomotor symptoms, and Hispanic women reported the most overall symptoms compared to Caucasian women [165]. Considering the finding that menopausal symptoms may be a key driver of body dissatisfaction during the menopausal transition [43], these global differences in the intensity and variety of menopausal symptoms experienced could influence the way menopausal women perceive body image across different cultures and regions.

Cultural views and attitudes towards menopause also fluctuate globally [166]. Across Western cultures, menopause is sometimes viewed as a “punishment” and a race against time due to its association with the alteration of physical appearance and the loss of youth, straying away from the thin and youthful beauty ideal [20,164]. It is also proposed that Western cultures tend to understand menopause as a medical condition to be addressed, whereas non-Western cultures tend to be more accepting of menopause as a natural part of the aging process [167]. While middle-aged women’s concerns over their changing appearance has been reported across several regions [140], one multi-ethnic qualitative study (*n* = 38) performed in the UK found that Muslim, Pakistani, African Caribbean, and West Indian midlife women were more concerned about physical changes related to their physicality and functionality (e.g., feeling tired, having joint pain, fear of becoming physically dependent on their children), compared to the British Caucasian women who generally expressed more concerns about their appearance, attractiveness, and sense of invisibility within society [157]. Non-Western traditional cultures typically hold more respect and less stigma towards older people, allowing women to see menopause and aging as a time of gaining social power and respect [22,164], which could reduce negative attitudes towards menopause and potentially alleviate the feelings of distress felt around aging-related physical changes. In contrast, a study of 75 in-depth interviews with Tunisian women in Tunisia, as well as Tunisian women and French women in France (45–70 years old), found that Tunisian working-class women living in both Tunisia and France experienced menopause with intense menopausal symptoms and strong feelings of social degradation, whereas Tunisian middle-class women experienced few menopausal symptoms but perceived a strong decline in their aesthetic and social value during menopause, and the French women seemed to report little change to their social value and few menopausal symptoms [168]. Such findings support the idea that the perceived loss in aesthetic and social value during menopause may vary by cultural setting and could in turn influence the body image of menopausal women differently across cultures.

Knowledge about menopause can also differ across cultures and potentially influence body image. While greater knowledge about menopause could help women feel more empowered during this transition, it appears that women living in cultures that bring little attention to menopause tend to experience fewer symptoms [162,164] and thus potentially experience less worry and distress during this stage. For instance, one study investigating Laotian and Australian women found that 57% of Australian women perceived the meaning of menopause as aging, and 43% had fears about weight gain, whereas Laotian women reported not knowing what menopause meant to them (81%) and not knowing about potential menopausal problems (85%) [169]. Similarly, a qualitative study of Macedonian women living in Australia showed that participants reported lacking information about menopause in their native language and showed highly variable knowledge of Hormonal Replacement Therapy (HRT) [156]. In certain low- and middle-income countries, it is also possible that the lack of knowledge and concern about menopause and body image comes from the fact that there are more pressing issues regarding women’s health in these areas. For example, a Sudanese physician interviewed as part of a 2021 clinical practice guide on menopause said that in his country, “there is no menopause”, stating that women are much more concerned with other issues like female circumcision [166].

Language may also influence global attitudes towards menopause. For example, among Arabic-speaking populations, the word menopause (*sin al ya’s*) is literally translated to “year of despair” or “year of no hope”, which could foster negative attitudes towards this stage of life [166]. It has also been reported that in certain Asian languages, there is no direct translation for the English meaning of “menopausal transition”. For instance, the Japanese and Chinese lay languages have words that translate to the narrow concept of the “end of menstruation” (*juejing* and *heikei*, respectively), as well as broader non-gender-specific terms that refer to the “transition between middle and old age” (*gengnianqi* and *könenki,* respectively) [170]. However, the Chinese term *gengnianqi* is typically viewed as a time of vulnerability to irritable outbursts, whereas the Japanese term *könenki* is commonly associated with bodily aches and self-restraint [170], highlighting how different languages can influence our perception of the meaning of the menopausal transition.

Finally, the intersecting experiences of immigrants can be highlighted through a qualitative study on body image in nine first-generation South Asian peri- or postmenopausal women living in Canada [142]. This study found that although South Asian women had fully adopted Western culture when it came to beauty standards and the internalization of the thin ideal, their South Asian traditions and practices remained influential in their lives as a way to cope with menopause, and they expressed a distrust for using Western medicine to alleviate their symptoms [142]. Such findings underscore the complexity of intersecting cultures and how different sociocultural factors can influence beauty standards, attitudes towards menopause, and coping strategies for dealing with the discomforts of the menopausal transition.

## 6. Key Findings and Future Research

As highlighted throughout this paper, there is limited research on the cultural determinants of body image during the menopausal transition. While the cross-cultural differences and sociocultural factors investigated in other age groups and during other reproductive milestones of women’s lives can help inform potential associations between cultural determinants and body image in menopausal women, there is a clear need for future studies to investigate how social and cultural factors interact to influence body image during menopause specifically. The complexity of social, biological, and psychological challenges that arise during this important transition make menopause a unique window of vulnerability for body image disturbances, which likely require the development of prevention and management strategies that are specific to this transition. In this review, the only identified studies investigating cultural or ethnic influences on body image in relation to the menopausal transition were qualitative studies. While qualitative research offers rich data that can help us understand the perceptions and experiences of menopausal women regarding their body image, it does not allow for the generalization of results or for the identification or larger trends regarding cultural and ethnic differences. Indeed, there is a dire need for more quantitative studies on this topic, including longitudinal studies tracking changes in body image across the menopausal transition, which could help provide a deeper understanding of the direction of associations between body image and menopause-related challenges [43]. Importantly, such studies should consider controlling for biological factors such as reproductive hormone levels and the severity of menopausal symptoms, as these factors may influence body image satisfaction [43]. The assessment of mental health problems such as depression and anxiety is also highly relevant considering their close relationship with body image [5]. In summary, future research should include both high-quality quantitative and qualitative studies to allow for a better understanding of the phenomenon of body dissatisfaction during the menopausal transition and its relationships with cultural determinants. This would support the elaboration of evidence-based and culturally sensitive interventions aimed at fostering a healthy body image in menopausal women across various cultures and regions of the world.

## 7. Conclusions

Cultural determinants of body image can greatly affect women’s body satisfaction in both similar and different ways across reproductive milestones like puberty, the perinatal period, and menopause. Several sociocultural factors such as societal beauty ideals and pressure from peers, family, romantic partners, and the media continue to negatively influence body image in middle-aged women, although there is limited information on their influence during the menopausal transition specifically. Moreover, this paper highlighted important research gaps when it comes to understanding the protective and detrimental role of different cultural norms, beliefs, and expectations in influencing body image satisfaction during the menopausal transition. Notably, the median duration of menopausal symptoms is over 7 years, including 4.5 years after the final menstrual period [45], underscoring the extent to which women’s lives can be affected by this complex physical, social, and psychological transition. Considering the crucial role of body satisfaction in predicting better mental health and quality of life in menopausal women [5,6], additional research is needed to characterize the social and cultural risk factors of body dissatisfaction during the menopausal transition and to better support middle-aged women through this natural yet complex transition.

## Data Availability

Not applicable.
